# Sarcopenia With High Subcutaneous Fat Predicts Metabolic Dysfunction Associated Steatotic Liver Disease Development After Pylorus Preserving Pancreaticoduodenectomy

**DOI:** 10.1002/wjs.12620

**Published:** 2025-05-15

**Authors:** Masashi Nakagawa, Naokazu Chiba, Shigeto Ochiai, Takahiro Gunji, Toru Sano, Koichi Tomita, Masatoshi Shigoka, Satoshi Tabuchi, Eiji Hidaka, Shigeyuki Kawachi

**Affiliations:** ^1^ Department of Digestive and Transplantation Surgery Tokyo Medical University Hachioji Medical Center Hachioji Japan

**Keywords:** MASLD, PPPD, sarcopenia, subcutaneous fat

## Abstract

**Background:**

MASLD after PPPD affects not only the long‐term prognosis but also the availability of postoperative adjuvant chemotherapy, its prevention and treatment are important issues. The aim of this study is to analyze whether objective preoperative factors could predict the development of postoperative MASLD in patients after PPPD.

**Methods:**

A total of 171 patients who underwent PPPD at our institute between April 2012 and May 2022 were retrospectively enrolled. We evaluated several CT findings, including various body fat areas and psoas muscles. The variables, including the CT findings and histopathological findings, were compared between the patients with and without MASLD after PPPD.

**Results:**

MASLD after PPPD developed 40 patients (23.4%). The clinical factors identified as predictive of postoperative MASLD on univariate analysis were sex (*p* < 0.001), subcutaneous fat area (*p* = 0.006), and psoas muscle area at the L3 level (*p* < 0.001) by univariate analysis. The multivariate analysis revealed subcutaneous fat area (odds ratio [OR] = 3.694, 95% confidence interval [CI]:1.315–10.377, *p* = 0.013) and psoas muscle area of the L3 level (OR = 0.223, 95%CI: 0.079–0.633, *p* = 0.005) as independent predictive factors for postoperative NAFLD.

**Conclusion:**

High subcutaneous fat accumulation and reduced psoas muscle area (sarcopenia) were identified as independent risk factors for the development of MASLD after PPPD.

## Graphical abstract

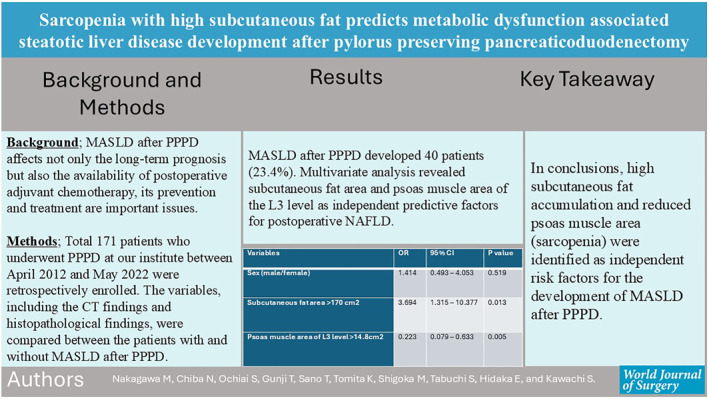

## 1 Introduction

Pylorus‐preserving pancreaticoduodenectomy (PPPD) is a common surgical treatment for periampullary diseases [1, 2]. Although the development of perioperative management has improved the long‐term survival following PPPD, long‐term nutrition following PPPD is an important factor influencing outcomes.

Metabolic dysfunction associated steatotic liver disease (MASLD) is a late complication of PPPD, which occurs in 8%–37% of patients [3]. MASLD is considered to be caused by malnutrition due to exocrine insufficiency of the residual pancreas. This condition is generally benign, and generally, it does not cause major problems for patients. However, in the long‐term, management may be necessary, as this condition can lead to malnutrition and decreases in patient quality‐of‐life [4]. Finally, as the development of MASLD after PPPD affects not only the long‐term prognosis but also the availability of postoperative adjuvant chemotherapy, its prevention and treatment are important issues.

Prediction of postoperative MASLD from preoperative factors is important, as predicting the postoperative exocrine function of the residual pancreas is difficult. Various predictive factors for postoperative MASLD have been reported; however, most are subjective, and no objective predictive factors have been identified [5, 6].

Therefore, this study aimed to analyze whether objective preoperative factors could predict the development of postoperative MASLD in patients after PPPD.

## 2 Patients and Methods

### 2.1 Patients

We retrospectively investigated the 180 patients who underwent PPPD for pancreatic head tumors at our institute between April 2012 and May 2022. Nine patients were excluded from this study because of a preoperative history of liver disease or death within 6 months postoperatively; therefore, clinicopathological data from 171 patients were evaluated. This study was approved by the Tokyo Medical University Ethics Committee (TS2023‐0446), and conducted in accordance with the provisions of the Declaration of Helsinki, as revised in Fortaleza, Brazil, in October 2013.

The surgical technique for PPPD in this study was performed as previously described [7, 8]. Delayed‐release pancreatic lipase supplementation (1200 mg/day) (LipaCreon, Eisai Co. Ltd., Tokyo, Japan) was initiated in all patients as soon as they were able to take oral nutrition, and continued indefinitely provided there were no signs of allergy or other problems.

### 2.2 Preoperative Radiological Analysis

**FIGURE 1:**
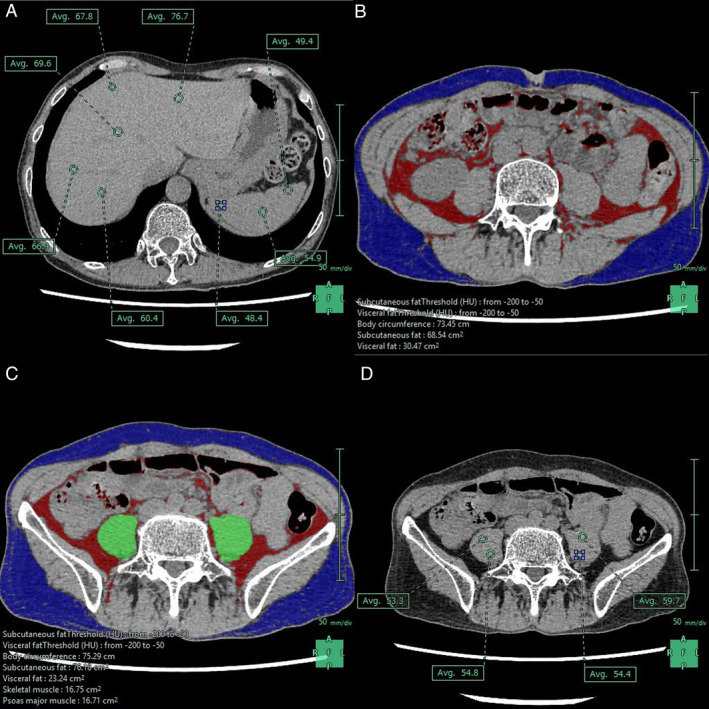
Evaluation of CT findings. (A) CT attenuation was measured at five locations in the liver and three locations in the spleen and the average value was calculated. (B) The areas of subcutaneous and visceral fat at the umbilical level were automatically calculated. (C) The psoas muscle area at the L3 level, which was added to the left and right psoas muscle areas, was also automatically calculated. (D) CT attenuation was measured at three locations on each side of the psoas muscle at the L3 level, and the average value was calculated.

All patients underwent preoperative abdominal CT during the arterial‐dominant, portal‐dominant, and equilibrium phases using Aquilion One (Toshiba Medical Systems), and light‐speed VCT (GE, Waukesha, WI, USA) and Revolution Flontier (GE, Waukesha, WI, USA), with images reconstructed at 1.25‐mm intervals as previously reported [7, 8]. Unenhanced and three‐phase contrast‐enhanced helical CT images were obtained. All CT data were analyzed using the Synapse Vincent software (Fuji Film). With this software, CT attenuation was measured at five locations in the liver and three locations in the spleen (Figure 1A), and the average value was calculated. The areas of subcutaneous and visceral fat at the umbilical level were automatically calculated using an abdominal fat analysis software (Figure 1B). The psoas muscle area at the L3 level, which was added to the left and right psoas muscle areas, was also automatically calculated using the muscle analysis software (Figure 1C). CT attenuation was measured at three locations on each side of the psoas muscle at the L3 level, and the average value was calculated (Figure 1D). All evaluations were performed by two investigators (M.N. and N.C., with 8 and 20 years of post‐training experience in surgery and CT imaging, respectively), and the average value was adopted.

### 2.3 Definition of MASLD

MASLD was defined as a liver‐to‐spleen attenuation ratio of less than 0.9, calculated after measurement of the average CT attenuation of the liver and spleen 6 months post‐operatively. The CT attenuation was measured using the method described above.

### 2.4 Statistical Analysis

All statistical analyses were performed using SPSS 28.0 for Windows (IBM Corp., Armonk, NY, USA). All variables were incorporated into the univariate analysis, and only those variables showing statistical significance (*p* < 0.05) were evaluated using univariate logistic analyses. Multivariate analysis using logistic regression was subsequently performed using factors with statistical significance in the univariate analysis. The performance of the predictive mode was assessed using receiver operating characteristic (ROC) curves and the corresponding areas under the curve (AUC) on the test data.

## 3 Results

### 3.1 Patients Characteristics

A total of 171 patients, including 95 males (55.2%) and 76 females (44.8%) with a median age of 72.0 years, were included in this study. The underlying diseases were as follows: 87 (50.9%) cases of pancreatic cancer, 18 (10.5%) cases of intraductal papillary mucinous neoplasm (IPMN) or intraductal papillary mucinous carcinoma (IPMC), 43 (25.1%) cases of bile duct cancer, 10 (5.8%) case of cancer of the papilla of vater, and 13 (7.7%) cases of other diseases. Preoperative chemotherapy was administered in 18 (10.5%) patients. All patients underwent PPPD, with a median operative time of 381 min, and a median blood loss of 360 mL. Further, 36 patients underwent additional combined vascular resection. Postoperative complications of Clavien–Dindo grade 3A were observed in 63 patients (36.8%) and postoperative pancreatic fistula (POPF) over grade B in 50 patients (29.2%).

### 3.2 Clinical Feature Predictive for Postoperative MASLD and Non‐MASLD Patients

The clinicopathological features stratified by postoperative MASLD are shown in Table 1. The *p*‐values between the two groups determined using univariate logistic regression analysis are shown for each factor. The clinical factors identified as predictive of postoperative MASLD on univariate analysis were sex (*p* < 0.001), subcutaneous fat area (*p* = 0.006), and psoas muscle area at the L3 level (*p* < 0.001). For subcutaneous fat area, the percentage of subcutaneous fat (defined as the subcutaneous fat area divided by the total fat area), and the subcutaneous fat index (defined as the subcutaneous fat area divided by body weight), were analyzed as factors in the univariate analysis, which also showed significant differences (data not shown). Moreover, univariate analysis was performed on the index of the psoas muscle at the L3 level (calculated as the psoas muscle area of the L3 level divided by the square of the height), revealing significant differences (data not shown). At postoperative 6 months, when MASLD was defined, there were no significant differences between the two groups in nutritional status or other biochemical tests.

**TABLE 1:** Patient characteristics stratified by postoperative MASLD.

Characteristic	MASLD group *N* = 40	Non‐MASLD group *N* = 131	*p* value
Sex (male/female)	11 (28%)/29 (72%)	84 (64%)/47 (36%)	< 0.001
Age (years)	69 (26–84)	72 (39–86)	0.138
Comorbidity			
Diabetes	12 (30%)	34 (26%)	0.614
Hypertension	13 (33%)	26 (20%)	0.061
Respiratory disease	1 (3%)	7 (5%)	0.456
Renal disease	8 (20%)	20 (15%)	0.072
Underlying disease			0.778
Pancreatic cancer	26 (64%)	61 (47%)	
IPMN or IPMC	2 (5%)	16 (12%)	
Bile duct cancer	10 (25%)	33 (25%)	
Cancer of the papilla of vater	1 (3%)	9 (7%)	
Others	1 (3%)	12 (9%)	
Background			
Body mass index (kg/m^2^)	20.2 (14.7–34.3)	21.1 (13.0–32.0)	0.931
Abdominal circumference (cm)	80.4 (59.8–103.6)	82.4 (59.1–104.1)	0.669
Preoperative laboratory data			
Platelet (× 10^3^/uL)	21.3 (6.2–53.9)	25.0 (13.8–47.1)	0.114
Lymphocyte count (/uL)	1556 (428–3368)	1479 (282–3059)	0.302
Albumin (g/dL)	3.5 (1.8–4.1)	3.5 (2.1–4.6)	0.491
PNI index	42.7 (1.4–60.3)	42.2 (22.1–50.8)	0.832
HbA1c (%)	6.1 (3.9–11.6)	6.2 (5.3–11.6)	0.527
Total cholesterol	170 (100–617)	172 (114–452)	0.982
Cholinesterase	241 (73–456)	263 (116–515)	0.082
GOT (U/L)	24 (11–39)	26 (11–82)	0.299
GPT (U/L)	22 (7–78)	30 (9–80)	0.107
C‐reactive protein (mg/dL)	0.2 (0.0–7.8)	0.2 (0.0–11.3)	0.841
Neoadjuvant chemotherapy	7 (18%)	11 (8%)	0.657
Preoperative radiological findings			
Volume of spleen (cm^3^)	104.8 (34.0–322.6)	122 (31.3–400.3)	0.247
Visceral fat area (cm^2^)	73.0 (50.3–226.8)	94.2 (20.5–383.6)	0.229
Subcutaneous fat area (cm^2^)	109.9 (60.35–391.24)	102.0 (40.5–215.0)	0.006
Psoas muscle area of L3 level (cm^2^)	14.4 (743.2–2622.4)	16.6 (547.8–3512.5)	< 0.001
CT attenuation of Psoas muscle (HU)	38.0 (17.6–57.1)	40.1 (13.6–58.7)	0.154
Intraoperative findings			
Blood loss (mL)	360 (20–1620)	355 (10–3615)	0.837
Operation time (min)	406 (223–580)	375 (130–573)	0.062
Combined vascular resection	15 (38%)	21 (16%)	0.516
Pancreatic stiffness (soft/hard)	13 (33%)/27 (67%)	41 (31%)/90 (69%)	0.153
Postoperative findings			
Complication > CD grade 3	13 (33%)	50 (38%)	0.516
POPF grade B/C	9 (23%)	41 (31%)	0.287
Adjuvant chemotherapy	16 (40%)	38 (29%)	0.197

Abbreviations: CD, Clavien–Dindo classification; CT, computed tomography; HU, Hounsfield unit; IPMC, intraductal papillary mucinous carcinoma; IPMN, intraductal papillary mucinous neoplasm; POPF, postoperative pancreatic fistula.

**FIGURE 2:**
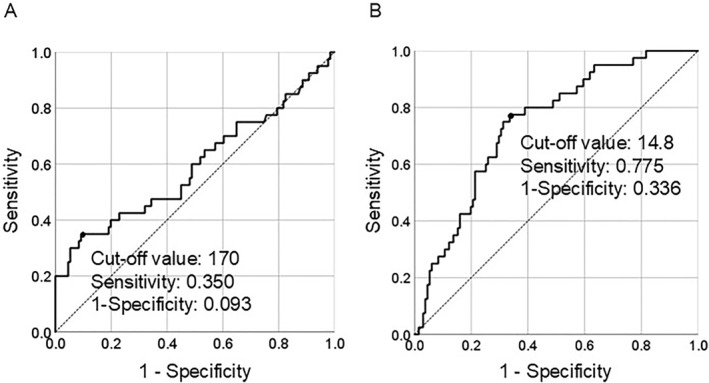
ROC curve analysis of subcutaneous fat area and psoas muscle area. The AUC was 0.593, and a cut‐off value of the subcutaneous fat area of 170 cm^2^ provided a sensitivity and specificity of 35.0% and 90.7%, respectively, at predicting MASLD (left panel). The area under the ROC curve was 0.742, and the cut‐off value of the psoas muscle area of L3 level of 14.8 cm^2^ provided a sensitivity and specificity of 77.5% and 66.4% for predicting MASLD (right panel).

The clinical significance of subcutaneous fat and psoas muscle areas at the L3 level were evaluated using ROC curve analysis (Figure 2). The AUC was 0.593, and a cut‐off value of the subcutaneous fat area of 170 cm^2^ provided a sensitivity and specificity of 35.0% and 90.7%, respectively, at predicting MASLD (Figure 2, left panel). The area under the ROC curve was 0.742, and the cut‐off value of the psoas muscle area of L3 level of 14.8 cm^2^ provided a sensitivity and specificity of 77.5% and 66.4% for predicting MASLD (Figure 2 right panel). Table 2 shows the results of the multivariate analysis of the three factors that showed significant differences in the univariate analysis. This analysis revealed subcutaneous fat area (odds ratio [OR] = 3.694, 95% confidence interval [CI]:1.315–10.377, *p* = 0.013) and psoas muscle area of the L3 level (OR = 0.223, 95%CI: 0.079–0.633, *p* = 0.005) as independent predictive factors for postoperative MASLD.

**TABLE 2:** Multivariate analysis of risk factors for postoperative MASLD.

Variables	OR	95% CI	*p* value
Sex (male/female)	1.414	0.493–4.053	0.519
Subcutaneous fat area > 170 cm^2^	3.694	1.315–10.377	0.013
Psoas muscle area of L3 level > 14.8 cm^2^	0.223	0.079–0.633	0.005

**FIGURE 3:**
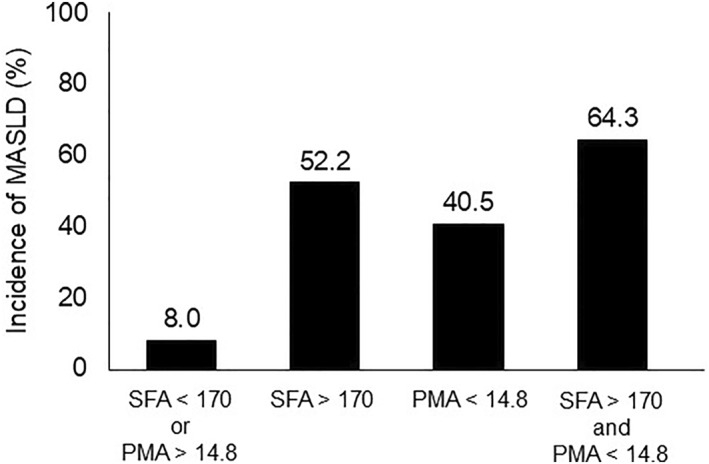
The incidence of postoperative MASLD in the four groups stratified by subcutaneous fat area and psoas muscle area. The incidence of postoperative MASLD was 8.0% in the group with subcutaneous fat area < 170 cm^2^ and psoas muscle area > 14.8 cm^2^, 52.2% in the group with subcutaneous fat area > 170 cm^2^, 40.5% in the group with psoas muscle area > 14.8 cm^2^, and 64.3% in the group with subcutaneous fat area > 170 cm^2^ and psoas muscle area < 14.8 cm^2^.

The incidence of postoperative MASLD in the four groups stratified by subcutaneous fat and psoas muscle areas at the L3 level is shown in Figure 3. The incidence of postoperative MASLD was 8.0% in the group with subcutaneous fat area < 170 cm^2^ and psoas muscle area > 14.8 cm^2^, 52.2% in the group with subcutaneous fat area > 170 cm^2^, 40.5% in the group with psoas muscle area > 14.8 cm^2^, and 64.3% in the group with subcutaneous fat area > 170 cm^2^ and psoas muscle area < 14.8 cm^2^.

## 4 Discussion

The present study identified preoperative subcutaneous fat area and psoas muscle area at the L3 level as significant predictive factors influencing the development of MASLD after PPPD. In this study, none of the preoperative nutritional status were significant predictors of MASLD. Also, on 6 postoperative months, there was no evidence of severe metabolic abnormalities with or without the development of MASLD. The advantages of the predictive factors in this study are that they are all objective preoperative factors that can be measured at any institution using data obtained from preoperative plain CT. Regarding previously identified predictive factors for MASLD development after PPPD, a recent study identified residual pancreatic CT attenuation values as correlated with acinar cell density, while residual pancreatic volume was found to predict MASLD [5]. However, the resection line of the residual pancreas and other factors cannot be determined preoperatively. In addition, CT attenuation values of the pancreas are difficult to measure in the presence of pancreatic atrophy, as it is difficult to pinpoint and select the residual pancreatic tissue. Furthermore, as has previously been noted, the residual pancreatic volume alone does not represent the exocrine function of the residual pancreas. Other research has reported that the pancreatic exocrine index calculated from the postoperative residual pancreatic volume and amylase activity of intraoperative pancreatic juice can predict MASLD, but not all of these predictive factors can be measured preoperatively. Furthermore, studies have reported that the residual pancreatic volume and residual pancreatic CT attenuation values can predict MASLD [6]. Although the mechanism underlying this association is unknown, there is no standardized method for measuring residual pancreatic CT attenuation values and volume [9]. However, it has been suggested that the residual pancreatic volume may have caused changes in fat metabolism. If all predictive factors can be measured preoperatively, then interventions could be introduced preoperatively to reduce the incidence of MASLD after PPPD.

Many studies have reported that an elevated visceral fat area is a cause of metabolic syndrome, a prognostic factor for various cancers, and a predictive factor for postoperative complications. However, very few reports have investigated elevated subcutaneous fat areas. The cutoff values for the subcutaneous fat area as a predictor of prognosis, postoperative complications, and metabolic syndrome in a few reports ranged from 98 to 194 cm^2^ [10, 11]. This study identified the subcutaneous fat area as an independent predictive factor for the development of MASLD after PPPD, in which the cutoff value was relatively high at 170 cm^2^, making it a reliable value. Moreover, several studies have reported that subcutaneous fat tissue has larger adipocytes [12] lower lipoprotein lipase activity [13] than other types of visceral fat tissue. Furthermore, high subcutaneous fat is associated with significantly lower bone strength, suggesting that subcutaneous fat is involved in the mechanism of bone resorption, which in turn may lead to sarcopenia [14]. The abnormal accumulation of subcutaneous fat may influence fat metabolism, triggering the development of MASLD after PPPD.

In the present study, the psoas muscle area was also identified as an independent predictor of the development of MASLD after PPPD. A great decrease in the psoas muscle area is defined as sarcopenia. Studies investigating the psoas muscle area to define sarcopenia often use the index value, which is the area divided by the square of the height, for the definition. The Index values has been reported to range from 3.5 to 6.5 cm^2^/m^2^ [15, 16]. Many studies also use these values for both sexes. In the present study, we identified a cutoff value of 14.8 cm^2^, which is an area rather than an index value. When this value was divided by the average height of 1.6 m, an index value of 5.7 cm^2^/m^2^ was identified, which, in the context of the literature, seems reasonable for defining sarcopenia.

Although several studies have reported a relationship between sarcopenia and the development of MASLD, no reports have investigated the relationship between sarcopenia and MASLD development after PPPD. A loss of muscle mass leads to insulin resistance, which is an important risk factor for MASLD [17]. Obesity, chronic low‐grade inflammation, vitamin D deficiency, lack of exercise, and hepatokinesis/myokinesis may also be involved in the pathophysiological mechanisms underlying sarcopenia and MASLD [18, 19]. However, there is insufficient data to support a direct causal relationship between sarcopenia and MASLD. Furthermore, it is currently difficult to conclude whether sarcopenia is a risk factor or a consequence of MASLD.

In the present study, abnormal subcutaneous fat accumulation and reduced psoas muscle area (sarcopenia) were identified as independent risk factors for the development of MASLD after PPPD. Therefore, preoperative intervention should include not only dietary weight loss, but also appropriate exercise therapy (aerobic and anaerobic exercise). However, this study has several limitations which should be considered. First, histopathological examination is insufficient for the diagnosis of postoperative MASLD. Further, liver biopsy was not performed due to the high invasivity of this procedure. Second, histopathological examination of subcutaneous adipocytes was not performed for the same reason. Third, since zinc was not measured in this study, either preoperatively or postoperatively, its relationship to zinc, one of the most important factors in the development of sarcopenia could not discussed. On the other hand, however, there was no association between sarcopenia development and postoperative pancreatic enzyme deficiency (data not shown). Fourth, the retrospective single center design of this study may have introduced selection bias. Future prospective studies are required to verify these results. In addition, further studies are required to assess the utility of the aforementioned nutritional and exercise therapy interventions. Additionally, it will be necessary to verify the mechanism by which abnormal subcutaneous fat accumulation and sarcopenia lead to the development of MASLD in vivo.

## Author Contributions

**Masashi Nakagawa:** conceptualization, data curation, formal analysis, investigation, methodology, project administration, resources, supervision, validation, visualization, writing – original draft, writing – review and editing. **Naokazu Chiba:** conceptualization, data curation, investigation, project administration, supervision, validation, writing – review and editing. **Shigeto Ochiai:** data curation. **Takayuki Gunji:** data curation. **Toru Sano:** data curation. **Koichi Tomita:** data curation. **Masatoshi Shigoka:** data curation. **Satoshi Tabuchi:** data curation. **Eiji Hidaka:** data curation. **Shigeyuki Kawachi:** conceptualization, data curation, writing – review and editing.

## Conflicts of Interest

The authors declare no conflicts of interest.
